# Serum Autoantibody Profiling of Patients with Paraneoplastic and Non-Paraneoplastic Autoimmune Retinopathy

**DOI:** 10.1371/journal.pone.0167909

**Published:** 2016-12-08

**Authors:** Josianne C. ten Berge, Joost van Rosmalen, Jacolien Vermeer, Cecilia Hellström, Cecilia Lindskog, Peter Nilsson, Ulrika Qundos, Aniki Rothova, Marco W. J. Schreurs

**Affiliations:** 1 Department of Ophthalmology, Erasmus University Medical Center, Rotterdam, The Netherlands; 2 Department of Biostatistics, Erasmus University Medical Center, Rotterdam, The Netherlands; 3 Department of Immunology, Erasmus University Medical Center, Rotterdam, The Netherlands; 4 Affinity Proteomics, SciLifeLab, School of Biotechnology, KTH Royal Institute of Technology, Stockholm, Sweden; 5 SciLifeLab, Department of Immunology, Genetics and Pathology, Uppsala University, Uppsala, Sweden; Carl von Ossietzky Universitat Oldenburg, GERMANY

## Abstract

**Purpose:**

Although multiple serum antiretinal autoantibodies (ARAs) have been reported in patients with paraneoplastic and non-paraneoplastic autoimmune retinopathy ((n)pAIR), not all retinal antigens involved in (n)pAIR are specified. This study aims to serologically identify patients with presumed (n)pAIR through determination of both known and unknown ARAs by autoantibody profiling.

**Methods:**

An antigen suspension bead array using 188 different antigens representing 97 ocular proteins was performed to detect ARAs in serum samples of patients with presumed (n)pAIR (n = 24), uveitis (n = 151) and cataract (n = 21). Logistic regressions were used to estimate the associations between ocular antigens and diagnosis. Validation of interphotoreceptor matrix proteoglycan 2 (IMPG2) and recoverin antigens was performed by immunohistochemistry and immunoblot, respectively.

**Results:**

Samples of patients with presumed (n)pAIR exhibited a broad spectrum of ARAs. We identified retinal antigens that have already been described previously (e.g. recoverin), but also identified novel ARA targets. Most ARAs were not specific for (n)pAIR since their presence was also observed in patients with cataract or uveitis. High titers of autoantibodies directed against photoreceptor-specific nuclear receptor and retinol-binding protein 3 were more common in patients with presumed (n)pAIR compared to uveitis (p = 0.015 and p = 0.018, respectively). The presence of all other ARAs did not significantly differ between groups. In patients with presumed (n)pAIR, anti-recoverin autoantibodies were the most prevalent ARAs. Validation of bead array results by immunohistochemistry (anti-IMPG2) and immunoblot (anti-recoverin) showed concordant results in (n)pAIR patients.

**Conclusions:**

Patients with (n)pAIR are characterized by the presence of a broad spectrum of ARAs. The diagnosis of (n)pAIR cannot be based on the mere presence of serum ARAs, as these are also commonly present in uveitis as well as in age-related cataract patients.

## Introduction

Paraneoplastic and non-paraneoplastic autoimmune retinopathy ((n)pAIR) is a rare blinding retinal disorder of unknown pathogenesis. It is presumed that antiretinal autoantibodies (ARAs) are involved in the pathogenesis of (n)pAIR and damage ocular tissue causing poor visual outcome. Symptoms associated with (n)pAIR are progressive visual loss (most often bilateral), visual field loss frequently associated with a ring scotoma or loss of the peripheral field, and decreased amplitudes on electroretinogram (ERG). [1–4]

Paraneoplastic autoimmune retinopathy (pAIR) includes two subgroups: cancer associated retinopathy (CAR) and melanoma associated retinopathy (MAR). In pAIR the presence of the same auto-antigens in both retinal tissue and malignant tissue has previously been described (e.g. recoverin). [5–7] The presence of ARAs however is not conclusive for the diagnosis of (n)pAIR, since several ARAs were also reported in patients with other ocular disorders and individuals without ocular disease. [8] Nevertheless, ARAs are considered to support the diagnosis of (n)pAIR, which is often difficult to confirm by clinical symptoms only.[9]

Multiple serum ARAs have regularly been reported in affected patients (Table 1), although not all retinal autoantibodies involved in the pathogenesis of (n)pAIR are known and information regarding their exact pathological roles is lacking. [10] Further, a gold standard for the determination of ARAs is missing. [11–13] The optimal approach for the determination and specification of ARAs is currently unknown. Different techniques, including indirect immunofluorescence, western blot and enzyme-linked immunosorbent assay (ELISA), have been used for the detection of ARAs; however, results and conclusions differ and cannot be reliably compared.

**Table 1:** Previously described antiretinal autoantibodies in serum of patients with paraneoplastic and non-paraneoplastic autoimmune retinopathy [1, 14, 15].

Antigen	Associated with			Location in retina	Size (kDa)
	CAR	MAR	npAIR		
Recoverin [16]	x	x	x	Inner segments and nuclei of photoreceptor cells, outer plexiform layer	23
α—Enolase [17]	x	x	x	Inner segments of the cone cells, Müller cells and ganglion cell layer	46
Carbonic anhydrase II [18]	x	x	x	Ganglion cell layer, inner nuclear layer, outer segments of photoreceptors	30
Heat shock cognate protein 70 [19]	x	x	x	N/A	65
Transducin α [20] (guanine nucleotide-binding protein G(t) subunit alpha-1)	x	x	x	Outer and inner segments of photoreceptor cells, cytoplasm of ganglion cells	40
Transducin β [21] (guanine nucleotide-binding protein G(I)/G(S)/G(T) subunit β-1)	x	x		Photoreceptor cells, ganglion cell layer	35
Arrestin (S-antigen) [22, 23]		x	x	Photoreceptor cells	48
Interphotoreceptor binding protein [24–26] (retinol binding protein 3)		x	x	Outer and inner segments of photoreceptor cells	141
Rhodopsin [27, 28]		x	x	Rod photoreceptor cells	40
Photoreceptor-cell-specific nuclear receptor [29]	x			Outer nuclear layer	44.7
Müller-cell-specific antigen [30]		x	x	N/A	35
Transient receptor potential cation channel subfamily M, member 1 [31–34]	x	x	x	Bipolar cells	182
Tubby-like protein 1 [35]	x		x	Photoreceptor cells	78
Bestrophin-1 [36]		x		Basal lateral membrane of retinal pigment epithelium	68
Aldolase A and C [15]		x	x	Ganglion cell layer, inner nuclear layer (aldolase C)	39
Glyceraldehyde 3-phosphate dehydrogenase [37]	x	x	x	Rod outer segments	30 and 36

Abbreviations: CAR: cancer associated retinopathy, MAR: melanoma associated retinopathy, npAIR: non-paraneoplastic autoimmune retinopathy

Currently, antigen bead arrays are being used to profile autoantibody reactivity in body fluids.[38] With this technique, very small volumes of body fluids can be tested for IgG reactivity across hundreds of samples towards hundreds of different antigens. This technique has already successfully been used for the analysis of autoantibodies in serum and cerebrospinal fluid. [39–41]

Our study aimed to serologically identify patients with presumed (n)pAIR through determination of ARAs. For this purpose, we used a bead array-based multiplex assay for autoantibody profiling using 188 ocular antigens representing 97 different retinal proteins.

## Methods

### Sample collection and patient selection

Serum samples were either collected during routine diagnostic analysis for the presence of anti-recoverin autoantibodies in the Laboratory of Medical Immunology of the Erasmus University Medical Center between April 2013 and August 2015 or were obtained from biobank of our department. The study was approved by the local ethical committee from the Erasmus University Medical Center (Medical Ethics Committee Erasmus MC) and adhered to the tenets of the Declaration of Helsinki. The ethical committee decided that no informed consent of patients was required for the use of the remainder of the diagnostic material, as the samples were anonymized and the patients were not subjected to additional risk or procedures. Samples which were obtained from the biobank (for which an approval of the ethical committee was obtained) included signed informed consent from all participants. All whole blood samples were centrifuged after at least 30’ clotting time at 3,000 rpm for 10 minutes, and serum was stored at -80°C.

According to the recently published report on the nomenclature of (n)pAIR, the general term autoimmune retinopathy (AIR) is recommended to indicate the non-paraneoplastic autoimmune retinopathy (npAIR) subtype. In our present series we indicate the specific subtype(s) of AIR (pAIR, npAIR or (n)pAIR) to prevent any misunderstanding regarding nomenclature.[9] The diagnosis of presumed (n)pAIR was made if the patients fulfilled all of the following inclusion criteria: 1. visual complaints, 2. markedly decreased amplitudes on ERG, 3. visual field loss, and 4. no alternative explanation for their ocular disorder. In addition, patients with genetically proven retinitis pigmentosa or a family history of retinitis pigmentosa were excluded. A total of 17 patients fulfilled the criteria indicated above and were included in this study. Patients fulfilling the criteria without a malignancy were indicated as presumed npAIR (N = 9), and patients with a malignancy were indicated as patients with presumed pAIR (N = 8).

An additional group of presumed pAIR (CAR or MAR) patients (N = 7) in whom ERG or visual field tests were not performed (choice of the patient or poor general condition), but who fulfilled all other inclusion criteria, was included separately. An additional required criterion for these patients comprised the development of a malignancy before or within 3 months after presentation with ocular problems.

We collected various clinical data of the patients with presumed (n)pAIR, including patient demographics (age and gender) and ocular characteristics such as complaints of photopsia, complaints of nyctalopia, subjective or objective problems with colour-vision, unilateral or bilateral visual problems and the presence of a malignancy in the medical history or during follow-up.

Controls consisted of two groups: 21 serum samples from cataract patients without retinal damage and 151 samples from patients with uveitis of different causes. Patients with age related cataract were included as controls rather than healthy people, as this disorders does not involve retina nor exhibits retinal damage, and represents a clinical setting in which the tests might be employed. Samples of control patients were collected at the department of Ophthalmology of the Erasmus University Medical Center between February 2009 and April 2015. Patient demographics (age and gender) and known malignancies of these patients were registered.

### Antigen suspension bead array

Autoantibody profiling was performed in all serum samples from patients with presumed (n)pAIR (n = 24), uveitis (n = 151) and cataract (n = 21). Antigens used for the autoantibody profiling were selected based on potential relevance to ocular diseases according to literature and previous positive retinal immunohistochemistry staining, resulting in 188 antigens (human protein fragments) representing 97 unique proteins. The protein fragments were produced within the Human Protein Atlas and designed to represent unique parts of each target protein.[42, 43] Protein fragments were 20–150 amino acids long (median 78 aa) and produced in *Escherichia coli*, with an affinity tag consisting of six histidines and an albumin binding domain from streptococcal protein G (His_6_ABP) (S1 Table). Immobilization onto color-coded magnetic beads was conducted as described previously [39]. In short, diluted antigens were covalently coupled to activated carboxy groups on color coded polystyrene beads (MagPlex, Luminex Corp.) by undirected amine coupling. In addition to the selected protein targets, one bead identity was used for immobilization of anti-human IgG (positive control), one for Epstein-Barr virus nuclear antigen 1 (second positive control), one for His_6_ABP (negative control, to monitor binding to the affinity tag) and one bead identity went through the coupling process without addition of antigen (second negative control, to monitor binding to bare beads). After incubation, the coupled beads were washed and stored in a blocking reagent before combining all bead identities to create a bead array in suspension. Samples were distributed across 96-well microtiter plates, together with triplicate aliquots of a sample pool and a buffer blank in each plate for determination of the intra- and inter-reproducibility. Serum samples were diluted 1:250 in assay buffer before being mixed with the bead array. Incubation was performed at room temperature for 2 hours followed by detection of the IgG reactivity by a fluorophore conjugated anti-human IgG Fab fragment and measured in a FlexMap3D instrument (Luminex Corp.).

### Recoverin immunoblot

For validation purposes, samples that tested positive for anti-recoverin autoantibodies on the antigen bead array, and all samples from patients with presumed (n)pAIR, were analysed on a recoverin specific immunoblot (Euroimmun AG, Lubeck, Germany). Membrane strips coated with recombinant human recoverin were incubated with a sample buffer for 5 minutes. After aspiration of the sample buffer, the membrane strips were incubated with diluted serum samples for 30 minutes on a shaking platform. Subsequently membrane strips were washed three times, incubated with secondary antibodies (enzyme conjugated anti-human IgG), washed again for three times and stained with a substrate solution which was capable of promoting an enzymatic colour reaction. To identify positive reactions, assessment of visible bands was performed relative to the included control. Results from the antigen suspension bead array and the recoverin specific immunoblot were compared.

### Immunohistochemistry of interphotoreceptor matrix proteoglycan 2 on human retina tissue

Another method for validation was performed with the antigens of interphotoreceptor matrix proteoglycan 2 (IMPG2). Polyclonal antibodies affinity purified against the IMPG2 antigens no. 214 and 205 were used as antigens for immunization of rabbits to generate polyclonal antibodies for immunohistochemistry on normal human tissues, in order to determine retina specificity and cell type expression. The antibodies were applied on tissue microarrays (TMAs) containing samples from 45 different human tissues, including retina from two individuals. TMAs from human tissues were generated essentially as previously described.[44] The TMAs contained 1 mm diameter formalin-fixed and paraffin-embedded tissue cores from 45 different histologically normal tissue types, including two samples of human eye: one male 75 years and one female 54 years. All samples were received from the Department of Pathology, Uppsala University Hospital, Sweden, approved by the local Research Ethics Committee (Uppsala, Sweden, Ups 02–577). Four-micrometer sections were cut from the TMA blocks, mounted on adhesive slides and baked at 60°C for 45 min. TMA slides were then deparaffinised in Neo-Clear^®^ (Merck Millipore, Darmstadt, Germany), followed by hydration in graded alcohols and blocking for endogenous peroxidase in 0.3% hydrogen peroxide. For antigen retrieval, slides were immersed and boiled in Citrate buffer^®^, pH6 (Lab Vision, Freemont, CA) for 4 min at 125°C and then allowed to cool to 90°C. Automated immunohistochemistry was performed essentially as previously described, using an Autostainer 480 instrument^®^ (Lab Vision).[44] Affinity purified polyclonal antibodies towards IMPG2 (HPA008779, antigen number 205, diluted 1:250 and HPA015907, antigen number 214, diluted 1:2500, both Atlas Antibodies AB) and a dextran polymer visualization system (UltraVision LP HRP polymer^®^, Lab Vision) were incubated for 30 min each at room temperature. Slides were developed for 10 min using Diaminobenzidine (Lab Vision) as chromogen. All incubations were followed by rinse in Wash buffer^®^ (Lab Vision) for 5 min. The slides were counterstained in Mayers hematoxylin (Histolab) and cover slipped using Pertex^®^ (Histolab) as mounting medium. Digital whole slide high-resolution images were captured with a 20× objective using an AperioScanScope XT Slide Scanner (Aperio Technologies, Vista, CA).

### Data analysis

Continuous variables were summarized using medians and ranges, and categorical variables were summarized using percentages. Patient demographics were compared between diagnosis groups using Mann Whitney U tests for continuous data and Fisher’s exact tests for categorical data. All data from the antigen suspension bead array were represented as ratios (antigen specific reactivity over patient background (represented by the His_6_ABP negative control bead)). A ratio of >2 was considered positive and a ratio of >25 was considered highly positive for the presence of ARAs. Logistic regressions with correction for age and gender were performed to analyse differences between the diagnosis groups ((n)pAIR versus uveitis and (n)pAIR versus cataract) for both ratio’s. In the logistic regression analyses, confidence intervals of the estimated odds ratios were calculated using a profile likelihood method, and the differences between groups were tested using a likelihood ratio test. To adjust for the multiple comparisons of the different antigens, a Bonferroni correction was used for the P-values of the logistic regression analyses, so that only P-values ≤ 0.0002 were considered statistically significant in these analyses. Intra- and inter-assay reproducibility was calculated with the coefficient of variation using the technical replicates within and between plates, based on the pooled serum samples.

The distribution of age, gender and the most prevalent ARAs (using the cut-off values for the ratio of 2 and 25) were compared between the subtypes of AIR (pAIR, npAIR) using Mann Whitney U tests for continuous data and Fisher’s exact tests for categorical data. The number of different ARAs per patient in highly positive titres were counted and compared between groups using a linear-by-linear association chi-square test. The association between the number of ARAs and age was assessed using Spearman’s rank correlation coefficient. All statistical tests were two-sided and used a significance level of 0.05. The analyses were performed using SPSS and R. [45]

## Results

### Patient characteristics

Characteristics of the patients with presumed (n)pAIR (N = 24) are specified in Table 2. The median age of patients was 67 years, with a range of 27–86 years. The majority of the patients were female (17/24, 71%). Most patients had bilateral visual complaints (21/24, 88%), and photopsia, nyctalopia and colour vision problems were noted frequently (12/19, 63%; 11/13, 85%; 9/11, 82%). A malignancy was seen in 15/24 (63%) patients (indicative for pAIR: CAR or MAR), of whom 8/24 (33%) patients had a malignancy in the past and 7/24 (29%) patients developed a malignancy during follow-up. The most frequently diagnosed malignancy was a lung carcinoma (6/15; 40%). A total of 9/24 (38%) patients did not have a malignancy and were diagnosed with presumed npAIR. Comparison of patient demographics (age and gender) between groups showed that patients with uveitis were significantly younger than patients with AIR (p<0.001). Gender did not differ between groups.

**Table 2:** General characteristics of patients.

Patient characteristics	(n)pAIR (N = 24)	Uveitis (N = 151)	Cataract (N = 21)
Gender (male-female)	7 (29%)–17 (71%)	63 (42%)– 88 (58%)*p = 0*.*271* †	10 (48%)– 11 (52%)*p = 0*.*233* †
Age in years (median; min-max)	67; 27–86	49; 17-86*p<0*.*001* †	69; 48-83*p = 0*.*339* †
Bilateral visual complaints	21/24 (88%)		
Complaints of photopsia	12/19 (63%) *		
Complaints of nyctalopia	11/13 (85%) *		
Colour-vision problems	9/11 (82%) *		
Presence of malignancy (pAIR) Malignancy in history Malignancy during follow-up	15/24 (63%) 8/24 (33%) 7/24 (29%)		

* Data not available for all patients

^†^ p-value of comparison with (n)pAIR patients

Abbreviations: (n)pAIR: non-paraneoplastic and paraneoplastic autoimmune retinopathy

### Antigen suspension bead array: highly positive titres of ARAs (ratio > 25)

**Fig 1:**
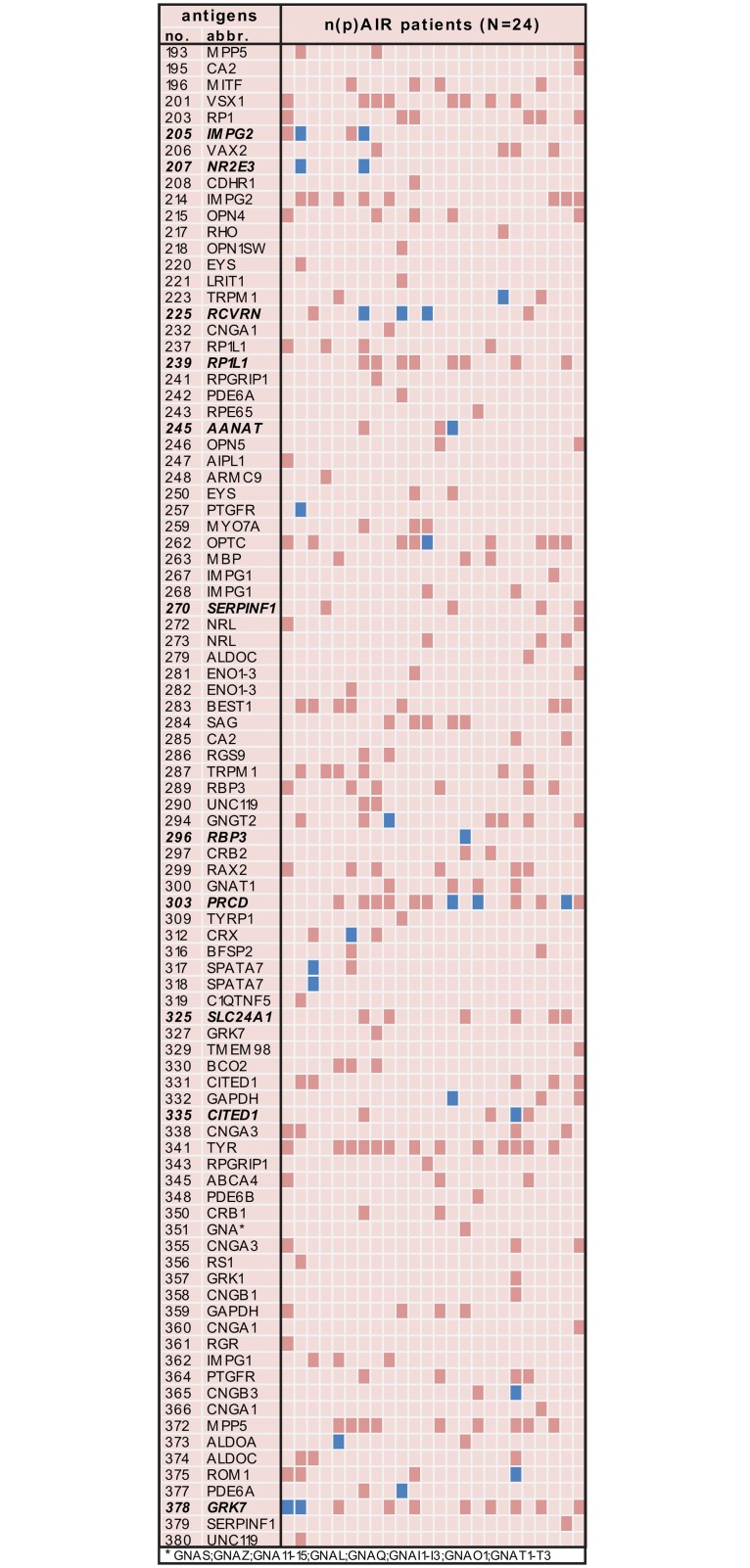
Spectrum of antiretinal autoantibodies in patients suspected of paraneoplastic and non-paraneoplastic autoimmune retinopathy. Blue: highly positive titer for the presence of antiretinal antibodies. Dark red: positive titer for the presence of antiretinal antibodies.

Patients with presumed (n)pAIR were characterized by the presence of a wide spectrum of ARAs (Fig 1 and S1 Fig). There was no specific ARA associated with a majority of patients with presumed (n)pAIR. In patients with presumed (n)pAIR, anti-recoverin autoantibodies were the most prevalent ARAs (12.5%). The presence of anti-recoverin autoantibodies was not fully specific for (n)pAIR, since high titres were also present in sporadic patients with cataract (4.8%; p = 0.351) or uveitis (1.3%; p = 0.061). Further, no association between the presence of anti-recoverin autoantibodies and a malignancy was found. High titre autoantibodies to photoreceptor-specific nuclear receptor and retinol-binding protein 3 were more prevalent in patients with (n)pAIR than in patients with uveitis (p = 0.015 and p = 0.018, respectively; p-values were not significant after applying correction for multiple testing). Autoantibodies towards IMPG2 (antigen number 205) were prevalent with highly positive titres in two patient samples with (n)pAIR (8.3%) and with lower prevalence in uveitis patients (2.0%). The results of the most prevalent ARAs present in high titres (ratio > 25) in patients with presumed (n)pAIR are shown in Table 3. The ARAs (in highly positive titres) indicated in Table 3 were only found in patients with presumed pAIR with the exception of two patients with presumed npAIR (one patients with npAIR was positive for high titres of antibodies against progressive rod-cone degeneration protein and one patient for high titres of Cbp/p300-interacting transactivator 1). The prevalence of high-titre ARAs (from Table 3), age and gender did not significantly differ between patients with npAIR and pAIR (all p > 0.05).

**Table 3:** Prevalence of antiretinal autoantibodies in paraneoplastic and non-paraneoplastic autoimmune retinopathy, uveitis and cataract*.

		Ratio > 25							Ratio >2						
		Prevalence of ARAs			(n)pAIR vs uveitis		(n)pAIR vs cataract		Prevalence of ARAs			(n)pAIR vs uveitis		(n)pAIR vs cataract	
Antigen number	Antigen	(n)pAIR	Uveitis	Cataract	OR; 2.5%–97.5%	p value	OR; 2.5%–97.5%	p value	(n)pAIR	Uveitis	Cataract	OR; 2.5%–97.5%	p value	OR; 2.5%–97.5%	p value
225	Recoverin †	12.5% (3/24)	1.3% (2/151)	4.8% (1/21)	6.3;0.91–54.7	0.061	2.98; 0.32–6.49	0.351	20.8% (5/24)	11.9% (18/151)	14.3% (3/21)	2.21;0.64–6.87	0.199	1.30; 0.25–7.63	0.753
303	Progressive rod-cone degeneration protein	12.5% (3/24)	8.6% (13/151)	14.3% (3/21)	2.01; 0.39–8.21	0.370	1.21; 0.19–8.23	0.837	50.0% (12/24)	34.4% (52/151)	57.1% (12/21)	1.83;0.73–4.58	0.195	0.73; 0.20–2.50	0.611
205	Interphotoreceptor matrix proteoglycan 2	8.3% (2/24)	2.0% (3/151)	0% (0/21)	3.82; 0.46–6.43	0.195	NA	0.062	16.7% (4/24)	25.2% (38/151)	19.0% (4/21)	0.59; 0.16–1.75	0.354	1.28; 0.244–6.95	0.766
207	Photoreceptor-specific nuclear receptor †	8.3% (2/24)	0% (0/151)	0% (0/21)	NA	0.015	NA	0.062	8.3% (2/24)	1.3% (2/151)	0% (0/21)	5.15; 0.55–49.67	0.141	NA	0.062
378	G protein-coupled receptor kinase 7	8.3% (2/24)	8.6% (13/151)	0% (0/21)	1.10; 0.16–4.77	0.905	NA	0.081	41.7% (10/24)	55.6% (84/151)	57.1% (12/21)	0.67; 0.26–1.69	0.399	0.47; 0.12–1.72	0.253
245	Serotonin N-acetyltransferase	4.2% (1/24)	0% (0/151)	0% (0/21)	NA	0.126	NA	0.251	12.5% (3/24)	0% (0/151)	4.8% (1/21)	NA	0.003	3.72; 0.39–84.11	0.262
296	Retinol-binding protein 3 †	4.2% (1/24)	0% (0/151)	0% (0/21)	NA	0.018	NA	0.272	4.2% (1/24)	2.6% (4/151)	4.8% (1/21)	2.65; 0.12–22.72	0.456	0.71; 0.03–19.31	0.815
335	Cbp/p300-interacting transactivator 1	4.2% (1/24)	0.6% (1/151)	0% (0/21)	7.07; 0.27–7.13	0.203	NA	0.377	16.7% (4/24)	6.0% (9/151)	9.5% (2/21)	6.52; 1.43–28.34	0.017	1.22; 0.17–10.56	0.842
239	Retinitis pigmentosa 1-like 1 protein	0% (0/24)	0.6% (1/151)	0% (0/21)	NA	0.537	NA	NA	33.3% (8/24)	15.2% (23/151)	23.8% (5/21)	3.54; 1.23–10.02	0.020	1.90; 0.47–8.75	0.375
325	Sodium / potassium / calcium exchanger 1	0% (0/24)	1.3% (2/151)	0% (0/21)	NA	0.674	NA	NA	25.0% (6/24)	17.9% (27/151)	4.8% (1/21)	1.41; 0.46–3.93	0.530	8.67; 1.13–188.88	0.037
270	Pigment epithelium-derived factor	0% (0/24)	0% (0/151)	0% (0/21)	NA	NA	NA	NA	16.7% (4/24)	10.6% (16/151)	0% (0/21)	1.64; 0.41–5.48	0.456	NA	0.030

* Calculation of OR was not possible in case of an ARA prevalence of 0 in either group

^†^ ARAs which have been described also in previous studies as autoantibodies associated with (n)pAIR

Abbreviations: (n)pAIR: non-paraneoplastic and paraneoplastic autoimmune retinopathy, ARAs: antiretinal antibodies, OR: odds ratio, NA: not available

The number of highly positive ARAs present in individual patients is shown in table 4. A higher number of different ARAs per patient was most prevalent in patients with presumed (n)pAIR and least present in patients with cataract. Three or more different ARAs were present in 29% of the patients with presumed (n)pAIR, compared to 24% of the patients with uveitis and 14% of the patients with cataract. The number of highly positive ARAs did not show any statistical differences between presumed (n)pAIR and uveitis (p = 0.457) or cataract (p = 0.385). Furthermore, there was no correlation between the number of ARAs and age (p = 0.926).

**Table 4:** Number of highly positive antiretinal autoantibodies per patient.

No. of highly positive ARAs (ratio > 25)	(n)pAIR (N = 24)	Uveitis (N = 151)	Cataract (N = 21)
0	37.5% (9/24)	45.7% (69/151)	52.4% (11/21)
1	33.3% (8/24)	29.8% (45/151)	33.3% (7/21)
2	12.5% (3/24)	13.2% (20/151)	4.8% (1/21)
3	12.5% (3/24)	7.9% (12/151)	4.8% (1/21)
4	4.2% (1/24)	2.6% (4/151)	0% (0/21)
5	0% (0/24)	0.6% (1/151)	4.8% (1/21)

Abbreviations: (n)pAIR: non-paraneoplastic and paraneoplastic autoimmune retinopathy, ARAs: antiretinal antibodies

### Antigen suspension bead array: positive titres of ARAs (ratio > 2)

The samples of patients with presumed (n)pAIR as well as both control cohorts exhibited a broad spectrum of positive ARAs (Fig 1 and S1 Fig). None of the ARAs were specific for presumed (n)pAIR only. Autoantibodies directed against serotonin N-acetyltransferase, cbp/p300-interacting transactivator 1 and retinitis pigmentosa 1-like 1 protein were more prevalent in patients with presumed (n)pAIR than in patients with uveitis (p = 0.003, p = 0.017 and p = 0.020; p-values were not significant after applying correction for multiple testing). When comparing the serum of patients with presumed (n)pAIR to the serum of patients with cataract, in presumed (n)pAIR autoantibodies directed against sodium/potassium/calcium exchanger 1 and pigment epithelium-derived factor were more often present (p = 0.037 and p = 0.030). The presence of most ARAs indicated in Table 3 was predominantly found in patients with presumed pAIR (CAR or MAR), but (often less frequently) also in patients with presumed npAIR. The prevalence of low-titre ARAs (from Table 3) was not significantly different between patients with npAIR and pAIR (p > 0.05).

The coefficient of variation based on replicates of the serum pools within and across plates (indicating the intra- and inter-reproducibility) ranged between 5 and 23% (median = 13%) for all 188 antigens. ARAs were present in all patients with presumed (n)pAIR and consequently all fulfilled the recent criteria for the diagnosis of (n)pAIR.[9]

### Recoverin immunoblot

Anti-recoverin autoantibodies on immunoblot were positive in 3 out of 24 (12.5%) patients with (n)pAIR. These positive results were in accordance with the positive high titre results on the antigen suspension bead array. Occasional discrepancy between the recoverin immunoblot and the antigen suspension bead array (using a high cut-off value, ratio > 25) was found in the controls (3 patients positive in antigen suspension bead array while negative on recoverin immunoblot).

### IMPG2 expression in human retina tissue

**Fig 2:**
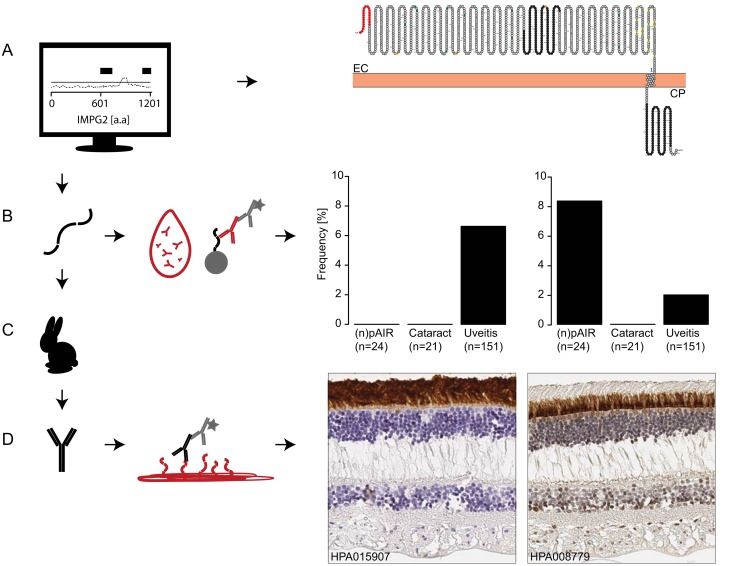
Screening and validation for interphotoreceptor matrix proteoglycan 2. (Left panel, A-D) The path from antigen design and generation to autoantibody screening in serum and secondly protein expression in retinal tissue. (A) Two antigens representing non-overlapping regions with either an extracellular or cytoplasmic location of IMPG2 were selected for recombinant protein expression (antigen 214 and 205 respectively, amino acids highlighted in black). (B) Detection of autoantibody reactivity with IMPG2 antigens using the antigen suspension bead array. Ratios > 25 are displayed per disease group for antigen 214 (left) and 205 (right). (C) The antigens were further used as antigens for immunization of rabbits to generate polyclonal antibodies. (D) Antibodies HPA015907 and HPA008779, affinity purified against antigens 214 and 205, were applied for immunohistochemical staining of human retina tissue. Both antibodies specifically showed cytoplasmic staining of cells in the photoreceptor layer in the retina (D, right). The antibody targeting the CP region of IMPG2 (HPA008779) stained only the inner segment of the photoreceptor layer, while HPA0015907 stained both inner and outer segment. EC; extracellular, CP; cytoplasmic, IMPG2; Interphotoreceptor matrix proteoglycan 2. Color annotation for central panel: black; Human Protein Atlas antigens and antibodies, red; human sample serum and tissue and grey; assay consumables suspension bead array and labelled detection antibodies.

The antigens towards IMPG2 (antigen number 214 and 205) represent two non-overlapping domains of IMPG2, located either extracellularly or in the cytoplasm (Fig 2A).[46] Antibodies directed against antigens 214 and 205, showed staining exclusively in cells in the photoreceptor layer of the retina. The antibodies targeting the cytoplasmic region of IMPG2 (against antigen number 205) stained only the inner segment of the photoreceptor layer, while HPA0015907 (antibodies against antigen number 214) stained both inner and outer segment (Fig 2D).

## Discussion

Our study shows that patients with (n)pAIR are characterized by the presence of a broad spectrum of various ARAs. We identified ARAs that have already been described in previous studies, such as anti-recoverin autoantibodies, but also identified new retinal targets. Our findings illustrate that serum ARAs are not only present in patients with (n)pAIR, but also in patients with cataract and uveitis. Though some ARAs appeared to be specific for (n)pAIR, their prevalence and consequently their sensitivity as markers for (n)pAIR were low. This autoantibody screening using 188 antigen provides insight into the autoimmune repertoire of patients with (n)pAIR and a base for further validation with independent methods for protein analysis and independent sample cohorts.

A gold standard for the determination of ARAs is currently lacking.[11] Different techniques are being used, hampering the comparison of results from various laboratories. [47] Moreover, the mere presence of ARAs does not provide any information on the role of this specific antibody.

In addition, information on clinical relevance of the specific ARAs and their pathological titres are lacking. A combination of different ARAs was observed in some cases and therefore their individual effects on retinal tissue could not be distinguished.

In our study, we performed statistical analyses using different cut-off levels. By using a high cut-off value, a ratio > 25, false positive results were minimized and retinal targets with a high specificity for (n)pAIR were found. The low cut-off value, a ratio of >2 (indicating ARAs with at least twice the reactivity of the negative control), was used for a more sensitive approach, limiting the exclusion of possibly relevant ARA targets with a lower titre. However, with both cut-off values, no ARAs were found eligible for diagnostic purposes. Some ARAs were specific for (n)pAIR, but had low prevalence while others were more frequently identified but lacked specificity.

In concordance with previous findings, positive results of serum anti-recoverin autoantibodies were not only observed in patients with presumed (n)pAIR, but also in patients with uveitis and cataract. Furthermore, no association between the presence of autoantibodies directed against recoverin and the presence of a malignancy was found. A discrepancy between the antigen suspension bead array results for anti-recoverin and the anti-recoverin immunoblot was found in three control patients (one with cataract and two with uveitis). The difference in results could be explained by the different techniques used for determination of ARAs imposing differences in analytical performance. Possibly, the number and/or availability of recoverin antigenic epitopes differed between the antigen suspension bead array and the immunoblot technique. The protein fragments we used in this study to screen for autoantibody reactivity in serum were designed to represent unique parts of each target protein. The binding of the autoantibodies towards their target may be influenced by the protein folding of antigens and may differ in comparison to full-length protein arrays or peptide arrays. Both linear and conformational epitopes, recognized by some ARAs, might be missed for some proteins, preventing recognition by certain autoantibodies.

The identification of new ARAs in (n)pAIR is in line with findings from previous studies using Western blot analysis for the determination of ARAs.[11, 48] Although many ARAs have already been identified, several studies have described so far unknown retinal autoantibodies presumably damaging retinal tissue and causing loss of vision.[14] In our study, we were able to identify novel ARAs possibly associated with (n)pAIR, e.g. serotonin N-acetyltransferase. Serotonin N-acetyltransferase plays a role in melatonin synthesis and is expressed only in the pineal gland and retina.[49] Autoantibodies directed against serotonin N-acetyltransferase have to our knowledge not been described in (n)pAIR so far. Another novel, although unspecific ARA found in this study is anti-G protein-coupled receptor kinase 7. Interestingly, it has been suggested previously that G protein-coupled receptor kinases in cancer cell lines are functionally associated with recoverin.[50] Moreover, protein IMPG2 was identified as an ARA and reactivity towards the cytoplasmic protein region (antigen number 205) was associated with (n)pAIR. Autoantibody reactivity towards a second antigen representing an extracellular region of IMPG2 was in contrast present in serum from uveitis patients. In short, ARAs targeting IMPG2 were identified using antigen arrays in serum samples and a retina specific protein expression of IMPG2 identified using immunohistochemistry in healthy human tissue.

Our present study focused on the autoantibodies prevalent in serum, which reflects systemic production and is probably not influenced by potential (additional) production or accumulation of specific autoantibodies within the eye. Analysis of local, intraocular retinal autoantibodies might show an entirely different pattern and may differ in clinical importance compared to retinal autoantibodies found in the peripheral circulation. The importance of locally produced autoantibodies has already been shown in cerebrospinal fluid for the central nervous system. In addition, it is unknown which autoantibodies penetrate from the circulation, through the blood retina barrier, into the retina and cause a local inflammation. Further research addressing the intraocular presence of specific retinal autoantibodies might elucidate the clinically relevant autoimmune processes directed against the retinal tissue in (n)pAIR.

A gold standard for the definitive diagnosis of (n)pAIR is currently lacking. Also in this study, the diagnosis of presumed (n)pAIR was based on clinical symptoms. To compensate for this inaccuracy, we used strict inclusion criteria and selected a uniform cohort of patients with unexplained visual loss, visual field defects and decreased or absent ERG while other diagnostic possibilities leading to this configuration of clinical characteristics were (so far as possible) excluded. The presence of ARAs was found in all our patients with presumed (n)pAIR and therefore all fulfilled the criteria for the diagnosis of (n)pAIR.[9]

Although the mere presence of ARAs supports the diagnosis of (n)pAIR, it has been stated that there are no specific ARAs which would be exclusive for (n)pAIR and none of the ARAs were identified to be of higher diagnostic value than other ARAs.[4, 9, 14] Our results are in full agreement with this statement. Proof for the definitive diagnosis of (n)pAIR is still missing and even the presence of ARAs is not specific for (n)pAIR, which has been illustrated by the finding of ARAs in control groups and healthy individuals.[8, 51, 52]

In conclusion, our study identified a heterogenous reactivity pattern of ARAs in serum of patients with (n)pAIR, although the presence of ARAs was not discriminatory between (n)pAIR, cataract and uveitis and exhibited a low sensitivity. Therefore, the diagnosis of (n)pAIR cannot be based on the mere presence of serum ARAs and such presence thus warrants careful interpretation. The determination of ARAs in intraocular fluid might provide more insight into the pathogenesis of (n)pAIR and might indicate more sensitive and specific diagnostic tools. Therefore, future research on the prevalence of ARAs in ocular fluid represents an important next step.

## Supporting Information

S1 Table Amino acid sequence and uniprot ID of ocular antigens used for the autoantibody profiling. (DOCX)

S1 Fig Spectrum of antiretinal autoantibodies in patients suspected of paraneoplastic and non-paraneoplastic autoimmune retinopathy, uveitis and cataract. ARAs not prevalent in patients suspected of paraneoplastic and non-paraneoplastic autoimmune retinopathy are not shown.* GNAS;GNAZ;GNA11-15;GNAL;GNAQ;GNAI1-I3;GNAO1;GNAT1-T3.
(EPS)
