# Supplementary material for: Serum Autoantibody Profiling of Patients with Paraneoplastic and Non-Paraneoplastic Autoimmune Retinopathy
Source: PLoS One. 2016 Dec 8;11(12):e0167909. doi: 10.1371/journal.pone.0167909 (PMC5145218; doi:10.1371/journal.pone.0167909)
Supplement: S1 Table — (DOCX) [file pone.0167909.s001.docx]

**S1. Table: Amino acid sequence and uniprot ID of ocular antigens used for the autoantibody profiling**

| **Antigen number** | **Antigen name** | **Short name** | **Uniprot** | **Antigen sequence (aa)** |
| --- | --- | --- | --- | --- |
| 193 | MAGUK p55 subfamily member 5 | MPP5 | Q8N3R9 | EDMRRRREEEGKKQELDLNSSMRLKKLAQIPPKTGIDNPMFDTEEGIVLESPHYAVKILEIEDLFSSLKHIQHTLVDSQSQEDISLLLQLVQNKDFQNAFKIHNAITVHMNKASPPFPLISNAQDLAQEVQTVLKPVHHKEGQELTAL |
| 194 | Melanocyte protein PMEL | PMEL | P40967 | PFSVSVSQLRALDRGNKHFLRNQPLTFALQLHDPSGYLAEADLSYTWDFGDSSGTLISRALVVTHTYLEPGPVTAQVVLQAAIPLTSCGSSPVPGTTDGHRPTAEAPNTTAGQVPTTEVVGTTPGQAPTAEPSGTTSCVRRAPPLSNN |
| 195 | Carbonic anhydrase 2 | CA2 | P00918 | YGKHNGPEHWHKDFPIAKGERQSPVDIDTHTAKYDPSLKPLSVSYDQATSLRILNNGHAFNVEFDDSQDKAVLKGGPLDGTYRLIQFHFHWGSLDGQGSEHTVDKKKYAAELHLVHWNTKYGDFGKAVQQPDGLAVLG |
| 196 | Microphthalmia-associated transcription factor | MITF | O75030 | HLLLRIQELEMQARAHGLSLIPSTGLCSPDLVNRIIKQEPVLENCSQDLLQHHADLTCTTTLDLTDGTITFNNNLGTGTEANQAYSVPTKMGSKLEDILMDDTLSPVGVTDPLLSSVSPGASKTSSRRSSMSMEETEHT |
| 197 | Tubulin-specific chaperone C | TBCC | Q15814 | PLPKKAEGDLGPSWVCGFSNLESQVLEKRASELHQRDVLLTELSNCTVRLYGNPNTLRLTKAHSCKLLCGPVSTSVFLEDCSDCVLAVACQQLRIHSTKDTRIFLQVTSRAIVEDCSGIQFAPYTWSYPEIDKDFESSGLDRSK |
| 198 | S-arrestin | SAG | P10523 | EPNHVIFKKISRDKSVTIYLGNRDYIDHVSQVQPVDGVVLVDPDLVKGKKVYVTLTCAFRYGQEDIDVIGLTFRRDLYFSRVQVYPPVGAASTPTKLQESLLKKLGSNTYPFLLTFPDYLPCSVMLQPAPQDSGKS |
| 199 | POU domain class 4, transcription factor 3 | POU4F3 | Q15319 | MSSVPCTSTSSTVPISHPAALTSHPHHAVHQGLEGDLLEHISPTLSVSGLGAPEHSVMPAQIHPHHLGAMGHLHQAMGMSHPHTVAPHSAMPACLSDVESDPRELEAFAERFKQRRIK |
| 200 | Fascin-2 | FSCN2 | O14926 | RVALKASNGRYVCMKKNGQLAAISDFVGKDEEFTLKLINRPILVLRGLDGFVCHHRGSNQLDTNRSVYDVFHLSFSDGAYRIRGRDGGFWYTGSHGSVCSDGERAEDFVFEFRE |
| 201 | Visual system homeobox 1 | VSX1 | Q9NZR4 | DSVLNSAEGGLLGSCAPWLLGMHKKSMGMIRKPGSEDKLAGLWGSDHFKEGSSQSESGSQRGSDKVSPENGLEDVAIDLSSSARQETKKVHPGAGAQGGSNSTALEGPQPGKVGA |
| 202 | Opsin-5 | OPN5 | Q6U736 | QVIDYKFACCQTGGLKATKKKSLEGFRLHTVTTVRKSSAVLEI |
| 203 | Oxygen-regulated protein 1 | RP1 | P56715 | VTCSPCEMCTVNKAYSPKETCNPSDTFFPSDGYGVDQTSMNKACFLGEVCSLTDTVFSDKACAQKENHTYEGACPIDETYVPVNVCNTIDFLNSKENTYTDNLDSTEELERGDDIQKDLNILTDPEYKNGFNTLVSHQNVSNLSSCG |
| 204 | Transducin beta-like protein 2 | TBL2 | Q9Y4P3 | SLKGQVLSTINTNQMNNTHAAVSPCGRFVASCGFTPDVKVWEVCFGKKGEFQEVVRAFELKGHSAAVHSFAFSNDSRRMASVSKDGTWKLWDTDVEYKKKQDPY |
| 205 | Interphotoreceptor matrix proteoglycan 2 | IMPG2 | Q9BZV3 | LQAHHDRSERESPFSGSSRQPDSLSSIENAVKYNPVYESHRAGCEKYEGPYPQHPFYSSASGDVIGGLSREEIRQMYESSELSREEIQERMRVLELYANDPEFAAFVR |
| 206 | Ventral anterior homebox 2 | VAX2 | Q9UIW0 | RRAESGGGGGRCGDRSGAGDLRADGGGHSPTEVAGTSASSPAGSRESGADSDGQPGPGEADHCRRILVRDAKGTIREIVLPKGLDLDRPKRT |
| 207 | Photoreceptor-specific nuclear receptor | NR2E3 | Q9Y5X4 | AETCAKLEPEDADENIDVTSNDPEFPSSPYSSSSPCGLDSIHETSARLLFMAVKWAKNLPVFSSLPFRDQVILLEEAWSELFLLGAIQWSLPLDSCPLLAPPEASAAGGAQGRLTLASMETRVLQETISRF |
| 208 | Cadherin-related family member 1 | CDHR1 | Q96JP9 | DMEGKYSVAEVFITLLDVNDHPPQFGKSVQKKTMVLGTPVKIEAIDEDAEEPNNLVDYSITHAEPANVFDINSHTGEIWLKNSIRSLDALHNITPGRDCLWSLEVQAKDRGSPSFSTTALLKIDITDAETLSRSPMAAFLIQTKD |
| 209 | Cadherin-related family member 1 | CDHR1 | Q96JP9 | ATVPVTIRIVDLNNHPPTFYGESGPQNRFELSMNEHPPQGEILRGLKITVNDSDQGANAKFNLQLVGPRGIFRVVPQTVLNEAQVTIIVENSAAIDFEKSKVLTFKLLAVEVNTPEKFSSTADV |
| 210 | L-dopachrome tautomerase | DCT | P40126 | NECDVCTDQLFGAARPDDPTLISRNSRFSSWETVCDSLDDYNHLVTLCNGTYEGLLRRNQMGRNSMKLPTLKDIRDCLSLQKFDNPPFFQNSTFSFRNALEGFDKADGTLDSQVMSLHNLVHSFLNGTNALPHSAANDPIFVVLHSFTDA |
| 211 | L-dopachrome tautomerase | DCT | P40126 | WSGPYILRNQDDRELWPRKFFHRTCKCTGNFAGYNCGDCKFGWTGPNCERKKPPVIRQNIHSLSPQEREQFLGALDLAKKRVHPDYVITTQHWLGLLGPNGTQPQFANCSVYDF |
| 212 | Carbonic anhydrase 4 | CA4 | P22748 | VTTKAKVDKKLGRFFFSGYDKKQTWTVQNNGHSVMMLLENKASISGGGLPAPYQAKQLHLHWSDLPYKGSEHSLDGEHFAMEMHIVHEKEKGTSRNVKEAQDPEDEIAVLA |
| 213 | Transducin beta-like protein 2 | TBL2 | Q9Y4P3 | RIRKEKPQQHNFTHRLLAAALKSHSGNISCMDFSSNGKYLATCADDRTIRIWSTKDFLQREHRSMRANVELDHATLVRFSPDCRAFIVWLANGDTLRVFKMTKRE |
| 214 | Interphotoreceptor matrix proteoglycan 2 | IMPG2 | Q9BZV3 | FDGGLGSGSGQKVDLITWPWSETSSEKSAEPLSKPWLEDDDSLLPAEIEDKKLVLVDKMDSTDQISKHSKYEHDDRSTHFPEEEPLSGPAVPIFADTAAESASLTLPKHISEVPGVDDYSVTKA |
| 215 | Melanopsin | OPN4 | Q9UHM6 | GWTHMEAAAVWGAAQQANGRSLYGQGLEDLEAKAPPRPQGHEAETPGKTKGLIPSQDPRM |
| 216 | Short-wave-sensitive opsin 1 | OPN1SW | P03999 | SRFIPEGLQCSCGPDWYTVGTKYRSESYT |
| 217 | Rhodopsin | RHO | P08100 | MNGTEGPNFYVPFSNATGVVRSPFEYPQYYLAEPWQFS |
| 218 | Short-wave-sensitive opsin 1 | OPN1SW | P03999 | RALKAVAAQQQESATTQKAEREVSRM |
| 219 | Regulator of G-protein signaling 9 | RGS9 | O75916 | LDAAQTHIYMLMKKDSYARYLKSPIYKDMLAKAIEPQETTKKSSTLPFMRRHLRSSPSPVILRQLEEEAKAREAANTVDITQPGQHMAPSPHLTVYTGTCMPPSPSSPFSSSCRSPRKPFASPSRFIRRPSTTIC |
| 220 | Protein eyes shut homolog | EYS | Q5T1H1 | INDCTSIPCMNEGFCQKSAHGFTCICPRGYTGAYCEKSIDNCAEPELNSVICLNGGICVDGPGH |
| 221 | Leucine-rich repeat, immunoglobulin-like domain and transmembrane domain-containing protein 1 | LRIT1 | Q9P2V4 | SETVISLIVTEPPTSTEHSGSPGALWARTGGGGEAAAYNNKLVARHVPQIPKPAVLATGPSVPSTKEELTLEHFQMDALGELSDGRAGPSEARMVRSVKVVG |
| 222 | Gamma-aminobutyric acid receptor subunit rho-1 | GABRR1 | P24046 | TTVQERKEQKLREKLPCTSGLPPPRTAMLDGNYSDGEVNDLDNYMPENGEKPDRMMVQLTLASERSSPQRKSQRSSYVSMRIDTHAIDK |
| 223 | Transient receptor potential cation channel subfamily M member 1 | TRPM1 | Q7Z4N2 | CEATYLLRQSSINSADGYSLYRYHFNGEELLFEDTSLSTSPGTGVRKKTCSFRIKEEKDVKTHLVPECQNSLHLSLGTSTSATPDGSHLAVDDLKNAEESKLGPDIGISKEDD |
| 224 | Transient receptor potential cation channel subfamily M member 1 | TRPM1 | Q7Z4N2 | VGGVNQDVEYSSITDQQLTTEWQCQVQKITRSHSTDIPYIVSEAAVQAEHKEQFADMQDEHHVAEAIPRIPRLSLTITDRNGMENLLSVKPDQTLGFPSLRSKSLHGHPRNVKSIQGKL |
| 225 | Recoverin | RCVRN | P35243 | SIYAKFFPDTDPKAYAQHVFRSFDSNLDGTLDFKEYVIALHMTTAGKTNQKLEWAFSLYDVDGNGTISKNEVLEIVMAIFKMITPEDVKLLPDDENTPEKRAEKIWKYFGKNDDDKLTEKEFIEGTLANKEILRLIQFEPQKVKEKMKN |
| 226 | Visual pigment-like receptor peropsin | RRH | O14718 | HVTLSIKHHTTSDCTESLNRDWSDQIDVTKMS |
| 227 | Rod cGMP-specific 3',5'-cyclic phosphodiesterase subunit alpha | PDE6A | P16499 | TLMESLTQFLGWSVLNPDTYESMNKLENRKDIFQDIVKYHVKCDNEEIQKILKTREVYGKEPWECEEEELAEILQAELPDADKYEINKFHFSDLPLTELELVKCGIQMYYELKVVDKFHIPQEALVRFMYSLSKGYRKITYHN |
| 228 | Unconventional myosin-VIIa | MYO7A | Q13402 | RYSGMMETIRIRRAGYPIRYSFVEFVERYRVLLPGVKPAYKQGDLRGTCQRMAEAVLGTHDDWQIGKTKIFLKDHHDMLLEVERDKAITDRVILLQKVIRGFKDRSNFLKLKNAATLIQRHWRGHNCRKNYGLMRLGF |
| 229 | Carbonic anhydrase 4 | CA4 | P22748 | GTQVNEGFQPLVEALSNIPKPEMSTTMAESSLLDLLPKEEKLRHYFRYLGSLTTPTCDEKVVWTVFREPIQLHREQILAFSQKLYYDKEQTVSMKDNVRPLQQLGQRTVIKSGA |
| 230 | LisH domain-containing protein ARMC9 | ARMC9 | Q7Z3E5 | HESELLGLVKEYLDFAEFEDTLKTFSKECKIKGKPLCKTVGGSFRDSKSLTIQKDLVAAFDNGDQKVFFDLWEEHISSSIRD |
| 231 | LisH domain-containing protein ARMC9 | ARMC9 | Q7Z3E5 | HIHFAIYLLKYSVGRPDKEELDEKISYFKTYLETKGAALSQTTEFLPFYALPFVPNPMVHPSFKELFQDSWTPELKLKLIKFLA |
| 232 | cGMP-gated cation channel alpha-1 | CNGA1 | P29973 | LSMKNNIINTQQSFVTMPNVIVPDIEKEIRRMENGACSSFSEDDDSASTSEESENENPHARGSFSYKSLRKGGPSQREQYLPGAIALFNVNNSSNKDQEPEEKKKKKKEKKSKSDNKNENKNDPEKKKKKKDKEKKKKEEKSKDKKE |
| 233 | Olfactomedin-like protein 2A | OLFML2A | Q68BL7 | PTSIPATTTTATTTPTPTTSLLPTEPPSGPEVSSQGREASCEGTLRAVDPPVRHHSYGRHEGAWMKDPAARDDRIYVTNYYY |
| 234 | Olfactomedin-like protein 2A | OLFML2A | Q68BL7 | LSEQLRHYENHSAIMLGIKKELSRLGLQLLQKDAAAAPATPATGTGSKAQDTARGKGKDISKYGSVQKSFADRGLPKPPKEKLLQVE |
| 235 | Olfactomedin-like protein 2A | OLFML2A | Q68BL7 | FYTVETVSSGTDCRCSCTAPPSSLNPCENEWKMEKLKKQAPELLKLQSMVDLLEGTLYSMDLMKVHAYVHKVASQMNTLEESIKANLSREN |
| 236 | Ventral anterior homebox 2 | VAX2 | Q9UIW0 | LNLSETQVKVWFQNRRTKQKKDQSRDLEKRASSSASEAFATSNILRLLEQGRLLSVPRAPSLLALTPSLPGLPASHRGTSLGDPRNSSPRLNPLSSASASPPLPPPLPAVCFSSAPLLDLPAGYELGSSAFEPYSWLERKVGS |
| 237 | Retinitis pigmentosa 1-like 1 protein | RP1L1 | Q8IWN7 | IWVSVLLKKTEKAFLAHLASAVAELRARWGLQDNDLLDQMAAELQQDVAQRLQDSTKRELQKLQGRAGRMVLE |
| 238 | Retinitis pigmentosa 1-like 1 protein | RP1L1 | Q8IWN8 | VPEVSRPMARRLSCSAGALITCLASLQLFEEDLGSPASKVRFKDSPWYQELLSISKDLWPGCDVGEDQ |
| 239 | Retinitis pigmentosa 1-like 1 protein | RP1L1 | Q8IWN9 | CYLCSDKKPPKTPSGPGRPQERNPTAQQLRDVEGQREAPGTSSSRKSLKTPRRILLIKNMDPRLQQTVVLSHRNTRN |
| 240 | Aryl-hydrocarbon-interacting protein-like 1 | AIPL1 | Q9NZN9 | LSNHEKMKAVPVLHGEGNRLFKLGRYEEASSKYQEAIICLRNLQTKEKPWEVQWLKLEKMINTLILNYCQCLL |
| 241 | X-linked retinitis pigmentosa GTPase regulator-interacting protein 1 | RPGRIP1 | Q96KN7 | MASPEVPIEAGQYRSKRKPPHGGERKEKEHQVVSYSRRKHGKRIGVQGKNRMEYLSLNILNGNTPEQVNYTEWKFSETNSFIGDGFKNQHEEEEMTLSHSALKQKEPLHPVNDKESSEQGSEVSEAQTTDSDDVIVPPMSQKYPKADSEK |
| 242 | Rod cGMP-specific 3',5'-cyclic phosphodiesterase subunit alpha | PDE6A | P16499 | EEVEKFLDSNIGFAKQYYNLHYRAKLISDLLGAKEAAVDFSNYHSPSSMEESEIIFDLLRDFQENLQTEKCIFNVMKKLCFLLQADRMSLFMYRTRNGIAELATRLFNVHKDAVLEDCLVMPDQEIVFPLDMGIVGHVAHSKKIAN |
| 243 | Retinoid isomerohydrolase | RPE65 | Q16518 | TKETWVWQEPDSYPSEPIFVSHPDALEEDDGVVLSVVVSPGAGQKPAYLLILNAKDLSEVARAEVEINIPVTFHGLFK |
| 244 | Retinoid isomerohydrolase | RPE65 | Q16518 | ETLETIKQVDLCNYVSVNGATAHPHIENDGTVYNIGNCFGKNFSIAYNIVKIPPLQADKEDPISKSEIVVQFPCSDRFKPSYVHSFGLTPNYIV |
| 245 | Serotonin N-acetyltransferase | AANAT | Q16613 | MSTQSTHPLKPEAPRLPPGIPESPSCQRRHTLPASEFRCLTPEDAVSAFEIEREAFISVLGVCPLYLDEIRH |
| 246 | Opsin-5 | OPN5 | Q6U736 | IAKVKSSSKEVAHFDSRIHSSHVLEMK |
| 247 | Aryl-hydrocarbon-interacting protein-like 1 | AIPL1 | Q9NZN9 | AEVWNEAEAKADLQKVLELEPSMQKAVRRELRLLENRMAEKQEEERLRCRNMLSQGATQPPAEPPTEPPAQSSTEPPAEPPTAPSAELSAGPPAEPATEPPPSPGHSLQH |
| 248 | LisH domain-containing protein ARMC9 | ARMC9 | Q7Z3E5 | VALLMNLCLRSTGKNMCAKVAGLVLKVLSDLLGHENHEIQPYVNGALYSILSVPSIREEARAMGMEDILRCFIKEGNAEMIRQIEFIIKQLNSEELPDGVLESD |
| 249 | Protein eyes shut homolog | EYS | Q5T1H1 | CECTSGWTGQNCSEEINECDSDPCMNGGLCHESTIPGQFVCLCPPLYTGQFCHQRYNLCDLLHNPCR |
| 250 | Protein eyes shut homolog | EYS | Q5T1H1 | FYIGGVSSLNLVNPMAIENEPVGFQGCIRQVIINNQELQLTEFGAKGGSNVGDCDGTACGYNTCRNGGECTVNGTTFSCRCLPDWAGNTCNQSVSCLNNL |
| 251 | Oxygen-regulated protein 1 | RP1 | P56715 | MSFPGRTESRSSGLKLAACSFSADVSPMERSSNQEGSLAEEINIQMTDQVAETCSSASWENATVDTDIIQGTQDQAKHRFYRPPTPGLRRVRQKKSVIGSVTLVSETEVQEKMIGQFSYSEERESGENKSEYHMFTHSCSKMSSVSN |
| 252 | Serotonin N-acetyltransferase | AANAT | Q16613 | FLTLCPELSLGWFEEGCLVAFIIGSLWDKERLMQESLTLHRSGGHIAHLHVLAVHRAFRQQGRGPILLWR |
| 253 | Peripherin-2 | PRPH2 | P23942 | GSLENTLGQGLKNGMKYYRDTDTPGRCFMKKTIDMLQIEFKCCGNNGFRDWFEIQWISNRYLDFSSKEVKDRIKSNVDGRYLVDGVPFSCCNPSSPRPCIQYQITNNSAHYSYDHQTEELNLWVR |
| 254 | Protein RD3 | RD3 | Q7Z3Z2 | SLISWLRWNEAPSRLSTRSPAEMVLETLMMELTGQMREAERQQRERSNAVRKVCTGVDYSWLA |
| 255 | Protein RD3 | RD3 | Q7Z3Z2 | QEEEAHKLTRQWSLRPRGSLATFKTRARISPFASDIRTISEDVERDTPPPLRSWSMPEFRAPK |
| 256 | Protein RD3 | RD3 | Q7Z3Z2 | RSTYDLSPIERLQLEDVCVKIHPSYCGPAILRFRQLLAEQEPEVQEVSQLFRSVLQEVLERMK |
| 257 | Prostaglandin F2-alpha receptor | PTGFR | P43088 | SMNNSKQLVSPAAALLSNTTCQTEN |
| 258 | Rod outer segment membrane protein 1 | ROM1 | Q03395 | AHYKDTEVPGHCQAKRLVDELQLRYHCCGRHGYKDWFGVQWVSSRYLDPGDRDVADRIQSNVEGLYLTDGVPFSCCNPHSPRPCLQNRLSDSYAHPLFDPRQPNQNLWA |
| 259 | Unconventional myosin-VIIa | MYO7A | Q13402 | WAVLTVQAYARGMIARRLHQRLRAEYLWRLEAEKMRLAEEEKLRKEMSAKKAKEEAERKHQERLAQLAREDAERELKEKEAARRKKELLEQMERARHEPVNHSDMVDKMFGFLGTSGGLPGQEGQAPSGFE |
| 260 | Melanocyte protein PMEL | PMEL | P40967 | PTAESTGMTPEKVPVSEVMGTTLAEMSTPEATGMTPAEVSIVVLSGTTAAQVTTTEWVETTARELPIPEPEGPDASSIMSTESITGSLGPLLDGTATLRLVKRQVPLDCVLYRYGSFSVTLDIVQGIESAEILQAV |
| 261 | Opticin | OPTC | Q9UBM4 | RIDLSNNLISSIDNDAFRLLHALQDLILPENQLEALPVLPSGIEFLDVRLNRLQSSGIQPAAFRAMEKLQFLYLSDNLLDSIP |
| 262 | Opticin | OPTC | Q9UBM4 | LPRKERKRREEQMPREGDSFEVLPLRNDVLNPDNYGEVIDLSNYEELTDYGDQLPEVKVTSLAPATSISP |
| 263 | Myelin basic protein | MBP | P02686 | ASTNSETNRGESEKKRNLGELSRTTSEDNEVFGEADANQNNGTSSQDTAVTDSKRTADPKNAWQDAHPADPGSRPHLIRLFSRDAP |
| 264 | Tubulin-specific chaperone C | TBCC | Q15814 | GLQKLINDSVFFLAAYDLRQGQEALARLQAALAERRRGLQPKKRFAFKTRGKDAASSTKVDAAPGIPPAVESIQDS |
| 265 | Tubulin-specific chaperone C | TBCC | Q15814 | MESVSCSAAAVRTGDMESQRDLSLVPERLQRREQERQLEVERRKQKRQNQEVEKENSHFFVATFVRERAAVEELLERAESVERL |
| 266 | Rhodopsin kinase | GRK1 | Q15835 | MDFGSLETVVANSAFIAARGSFDGSSSQPSRDKKYLAKLKLPPLSKCESLRDSLSLEFESVCLEQPIGKKLFQQFLQSA |
| 267 | Interphotoreceptor matrix proteoglycan 1 | IMPG1 | Q17R60 | NERTEEAECRCKPGYDSQGSLDGLEPGLCGPGTKECEVLQGKGAPCRLPDHSENQAYKTSVKKFQNQQNNKVISKRNSELLTVEYEEFNHQDWEGN |
| 268 | Interphotoreceptor matrix proteoglycan 1 | IMPG1 | Q17R60 | DSNKIESEEVYHGTMEEDKQPEIYLTATDLKRLISKALEEEQSLDVGTIQFTDEIAGSLPAFGPDTQSELPTSFAVITEDATLSPELPPVEPQLE |
| 269 | Bestrophin-1 | BEST1 | O76090 | SQVRRKTVEFNLTDMPEIPENHLKEPLEQSPTNIHTTLKDHMDPYWALENRSVLHLNQGHCIALCPTPASLALSLPFLHNFLGFHHCQSTLDLRPALAWGIYLATFTGILGKCSGPFLTSPWYHPEDFLGPGE |
| 270 | Pigment epithelium-derived factor | SERPINF1 | P36955 | KVPVNKLAAAVSNFGYDLYRVRSSMSPTTNVLLSPLSVATALSALSLGAEQRTESIIHRALYYDLISSPDIHGTYKELLDTVTAPQKNLKSASRIVFEKKLRIKSSFVAPLEKSYGTRPRVLTGNPRLDLQEINNWVQAQMKGKL |
| 271 | Tyrosinase | TYR | P14679 | FPRACVSSKNLMEKECCPPWSGDRSPCGQLSGRGSCQNILLSNAPLGPQFPFTGVDDRESW |
| 272 | Neural retina-specific leucine zipper protein | NRL | P54845 | FSEPGMVGATEGTRPGLEELYWLATLQQQLGAGEALGLSPEEAMELLQGQGPVPVDGPHGYYPGSPEETGA |
| 273 | Neural retina-specific leucine zipper protein | NRL | P54845 | RERDLYKARCDRLTSSGPGSGDPSHLFL |
| 274 | Retinol dehydrogenase 8 | RDH8 | Q9NYR8 | GQTLTVAQLDVCSDESVAQCLSCIQGEVDVLV |
| 275 | Long-wave-sensitive opsin 1 / Medium-wave-sensitive opsin 1 | OPN1LW; OPN1MW; OPN1MW2 | P04000 / P04001 | ISIVNQVSGYFVLGHPMCVLE |
| 276 | Prostaglandin F2-alpha receptor | PTGFR | P43088 | IQASRTWCFYNTEDIKDWEDRF |
| 277 | Heat shock cognate 71 kDa protein | HSPA8 | P11142 | ITKLYQSAGGMPGGMPGGFP |
| 278 | Age-related maculopathy susceptibility protein 2 | ARMS2 | P0C7Q2 | LSSSVVPVSFISTLRESVLDPGVGGEGASD |
| 279 | Fructose-bisphosphate aldolase C | ALDOC | P09972 | QASALNAWRGQRDNAGAATEEFIKRAEVNGLAAQGKYEGSGEDGGAAAQSLYIANHAY |
| 280 | Alpha-enolase | ENO3; ENO2; ENO1 | P06733 | QACKLAQENGWGVMVSHRSGETEDTFIADLVVGLCTGQIKTGAPCRSERLAKYNQLMRIEEE |
| 281 | Alpha-enolase | ENO3; ENO2; ENO1 | P06733 | LKNVIKEKYGKDATNVGDEGGFAPNIL |
| 282 | Alpha-enolase | ENO3; ENO2; ENO1 | P06733 | TSKGLFRAAVPSGASTGIYEALELRDNDKTRY |
| 283 | Bestrophin-1 | BEST1 | O76090 | EMHQDLPRMEPDMYWNKPEPQPPYTAASAQFRRASFMGSTFNISLNKEEMEFQPNQEDEEDAHAGIIGRFLGLQSH |
| 284 | S-arrestin | SAG | P10523 | TVSGFLGELTSSEVATEVPFRLMHPQPEDPAKESYQDANLVFEEFARHNLKDAGEAEEGKRDKNDVDE |
| 285 | Carbonic anhydrase 2 | CA2 | P00918 | PISVSSEQVLKFRKLNFNGEGEPEELMVDNWRPAQPLKNRQIKASFK |
| 286 | Regulator of G-protein signaling 9 | RGS9 | O75916 | VFARLSPKCPAVSHGRVQPLGDVGQQLPRLKSKRVANFFQIKMDVPTGSGTCLMDSEDAGTGESGDRATEKEVICPWE |
| 287 | Transient receptor potential cation channel subfamily M member 1 | TRPM1 | Q7Z4N2 | NAEESKLGPDIGISKEDDERQTDSKKEETISPSLNKTDVIHGQDKSDVQNTQLTVETTNIEGTISYPLEETKITRYFPDETINACKTMKSRSFVYSR |
| 288 | P Protein | OCA2 | Q04671 | PDQGKLWQLLALSPLENYSVNLSSHVDSTLLQVDLAGALVASGPSRPGREEHIVVELTQA |
| 289 | Retinol-binding protein 3 | RBP3 | P10745 | GVEPDITVPMSEALSIAQDIVALRAKVPTVLQTAGKLVADNYASAELGAKMATKLSGLQSRYSRVTSEVALAEILGADLQMLSGDPHLKAAHIPENAKDR |
| 290 | Protein unc-119 homolog A | UNC119 | Q13432 | GPLQRKQPIGPEDVLGLQRITGDYLCSPEENIYKIDFVRFKI |
| 291 | Retinaldehyde-binding protein 1 | RLBP1 | P12271 | TTKDHGPVFGPCSQLPRHTLQKAKDELNEREETREEAVRELQEMVQAQAASGEELAVAVAERVQEKDSGFFLRFIRARKFNVGRAYELLRGYVNFRL |
| 292 | Guanine nucleotide-binding protein G(T) subunit gamma-T1 | GNGT1 | P63211 | DQLKKEVTLERMLVSKCCEEVRDYV |
| 293 | Melanoma antigen recognized by T-cells 1 | MLANA | Q16655 | RRRNGYRALMDKSLHVGTQCALTRRCPQEGFDHRDSKVSLQEKNCEPVVPNAPPAYEKLSAEQSPPPYSP |
| 294 | Guanine nucleotide-binding protein G(I)/G(S)/G(O) subunit gamma-T2 | GNGT2 | O14610 | EQLKKEVKNTRIPISKAGKEIKEYVEAQAGNDP |
| 295 | Retinaldehyde-binding protein 1 | RLBP1 | P12271 | PARFKAIHFIHQPWYFTTTYNVVKPFLKSKLLERVFVHGDDLSGFYQEIDENILPSD |
| 296 | Retinol-binding protein 3 | RBP3 | P10745 | EDNIGYLRFDMFGDGELLTQVSRLLVEHIWKKIMHTDAMIIDMRFNIGGPTSSIPILCSYFFDEGPPVLLDKIYSRPDDSVSELWTHAQVVGERYGSK |
| 297 | Protein crumbs homolog 2 | CRB2 | Q5IJ48 | RWDDGLRHLVMLSFGPDQLQDLGQHVHVGGRLLAADSQPWGGPFRGCLQDLRLDGCHLPFFPLPLDNSSQPSELGGRQSWNLT |
| 298 | Retbindin | RTBDN | Q9BSG5 | PLQARSQQHHGLAADLGKGKLHLAGPCCPSEMDTTETSGPGNHPERCGVPSPECESFLEHLQRALRSRFR |
| 299 | Retina and anterior neural fold homeobox protein 2 | RAX2 | Q96IS3 | ERLESGSGAVAAPRLPEAPALPFARPPAMSLPLEPWLG |
| 300 | Guanine nucleotide-binding protein G(T) subunit gamma-T1 | GNAT1 | P63211 | SARQDDARKLMHMADTIEEGT |
| 301 | Retbindin | RTBDN | Q9BSG5 | MDEALETQLKTSRGRFSATESLPTLELLSQVDMDCRVHMRPIG |
| 302 | Protein crumbs homolog 2 | CRB2 | Q5IJ48 | IPAATFGLGGAPSSASFLLQELPGPNLTVSFLLRTRESAGLLLQFANDSAAGLTVFLSEGRIRAEAPGSPAVVLPG |
| 303 | Progressive rod-cone degeneration protein | PRCD | Q00LT1 | ANRVQPEPSDVDGAARGSSLDADPQSSGREKEPLK |
| 304 | Protein eyes shut homolog | EYS | Q5T1H1 | EGWLKVDDHKNKSIIAPGRLVGLNVFSQFYVGGYSEYTPDLLPNGADFKNGFQGCIFTLQVRTEKDGHFRGLGNPEGHPNAGRSVGQCHASPC |
| 305 | ADP-ribosylation factor-like protein 3 | ARL3 | P36405 | KLNVWDIGGQRKIRPYWKNYFENTDILIYVIDSADRKRFEETGQELAELLEEEKLSCVPVLI |
| 306 | Protein eyes shut homolog | EYS | Q5T1H1 | EQFSGPFCEVSAKPCVSLLFWKRGICPNSSSAYTYECPKGSSSQNGETDVSEFSLVPCQNGTDCIKISNDVMCICSPIFTDLLC |
| 307 | P Protein | OCA2 | Q04671 | GLAELVAGKRRLPRGAGGADPSHSCPRGAAGQSSWAPAGQEFASFLTKGRSHSSLPQMSSSRSKDSCFTENTPLLRNSLQEKGSRC |
| 308 | P Protein | OCA2 | Q04671 | DALGSRWRRPQQVTHNWTVYLNPRRSEHSVMSRTFEVLTRETVSISIRASLQQTQAVPLLMAHQYLRGSVD |
| 309 | 5,6-dihydroxyindole-2-carboxylic acid oxidase | TYRP1 | P17643 | VCDICTDDLMGSRSNFDSTLISPNSVFSQWRVVCDSLEDYDTLGTLCNSTEDGPIRRNPAGNVARPMVQRLPEPQDVAQCLEVGLFDTPPFYSNSTNSFRNTVEGYSDPTGKYDPAVRSLH |
| 310 | RPE-retinal G protein-coupled receptor | RGR | P47804 | IAWGRYHHYCTRSQLAWNSAV |
| 311 | Cone-rod homeobox protein | CRX | O43186 | PLPEAQRAGLVASGPSLTSAPYAMTYAPASAFCSSPSAYGSPSSYFSGLDPYLSPMVPQLGGPALSPLSGPSVGPSLAQSPTSLSGQSYGAYSPVDSLEFK |
| 312 | Cone-rod homeobox protein | CRX | O43186 | KARPAKRKAGTSPRPSTDVCPDPLGISDSYSPPLPGPSGSPTTAVATVSI |
| 313 | Cadherin-related family member 1 | CDHR1 | Q96JP9 | LVPEDIPAGSIIFKVHAVDRDTGSGGSVTYFLQNLHSPFAVDRHSGVLRLQAGATLDYERSRTHYITVVAKDGGGRLHGADV |
| 314 | Alpha-crystallin A chain | CRYAA | P02489 | KHNERQDDHGYISREFHRRYRLPSNVDQSALSCSLSADGMLTFCGPKIQTGLDATHAERAIPVSREEKPTSA |
| 315 | Alpha-crystallin A chain | CRYAA | P02489 | PSRLFDQFFGEGLFEYDLLPFLSSTISPYYRQSLFRTVLDSGISEVRSDRDKFVIFLDVKHFSPEDLTVKVQDDFVE |
| 316 | Phakinin | BFSP2 | Q13515 | LSRNYEEDVKLLHKQLAGCELEQMDAPIGTGLDDILETIRIQWERDVEKNRVEAGALLQAKQQAEVAHMSQTQEEKLAAALRVE |
| 317 | Spermatogenesis-associated protein 7 | SPATA7 | Q9P0W8 | RHLLHVLKVDLGCTSEENSVKQNDVDMLNVFDFEKAGNSEPNELKNESEVTIQQERQQYQKALDMLLSAPKDENEIFPSPTEFFMPIYKSKHSEGV |
| 318 | Spermatogenesis-associated protein 7 | SPATA7 | Q9P0W8 | QRIEAETQTELSFKSELGTAETKNMTDSEMNIKQASNCVTYDAKEKIAPLPLEGHDSTWDEIKDDALQHSSPRA |
| 319 | Complement C1q tumor necrosis factor-related protein 5 | C1QTNF5 | Q9BXJ0 | ECSVPPRSAFSAKRSESRVPPPSDAPLPFDRVLVNEQGHYDAVTGKFTCQV |
| 320 | POU domain class 4, transcription factor 3 | POU4F3 | Q15319 | HQGLEGDLLEHISPTLSVSGLGAPEHSVMPAQIHPHHLGAMGHLHQAMGMSHPHTVAPHSAMPACLSDVESDPR |
| 321 | Melanopsin | OPN4 | Q9UHM6 | PKYRVAIAQHLPCLGVLLGVSRRHSRPYPSYRSTHRSTLTSHTSNLSWISIRRRQESLGSESEV |
| 322 | Cyclic nucleotide-gated cation channel beta-1 | CNGB1 | Q14028 | LRSNNKPKEEKSVLILPPRAGTPKLFNAALAMTGKMGGKGAKGGKLAHLRARLKELAALEAAAKQQELVEQAKSSQDVKGEEGSAAPDQHTHPKE |
| 323 | Retinal-specific ATP-binding cassette transporter | ABCA4 | P78363 | THPERIAGRGIRIRDILKDEETLTLFLIKNIGLSDSVVYLLINSQVRPEQFAHGVPDLALKDIACSEALLERFIIFSQ |
| 324 | Beta,beta-carotene 9',10'-oxygenase | BCO2 | Q9BYV7 | VDFLPVMVHRLPVFKRYMGNTPQKKAVFGQCRGLPCVAPLLTTVEEAPRGISARVWGHFPKWLNGSLLRIGPGKFEFGKDKYNHW |
| 325 | Sodium / potassium / calcium exchanger 1 | SLC24A1 | O60721 | SSHQPIKLASRDLSSEEMMMMSSSPSKPSSEMGGKMLVPQASVGSDEATLSMTVENIPSMPKRTAKMIPTTTKNNYSPTAAGTERRKED |
| 326 | Bestrophin-2 | BEST2 | Q8NFU1 | RRLSFLLRKNSCVSEASTGASCSCAVVPEGAAPECSCGDPLLDPGLPEPEAPPPAGPEPLTLIPGPVEPFSIVTMP |
| 327 | G protein-coupled receptor kinase 7 | GRK7 | Q8WTQ7 | EEGPTKDSALQGLVATCASAPAPGNPQPFLSQAVATKCQAATTEEERVAAVTLAKAEAMAFLQEQPFKDFVTSAFYDKFLQWKLFEMQPVSD |
| 328 | Sodium / potassium / calcium exchanger 1 | SLC24A1 | O60721 | FDSTPTFLTHEVEANVLTSPRSVMEKNNLFPPRRVESNSSAHPWGLVGKSNPKTPQGTVLLHTPATSEGQVTISTMTGSSPAETKAFTAAWSLRNPSPRTSVSAIKT |
| 329 | Transmembrane protein 98 | TMEM98 | Q9Y2Y6 | CRQRYCRPRDLLQRYDSKPIVDLIGAMETQSEPSELELDDVVITNPHIEAILENEDWIEDASGLMSHCIAILKICHTLTEKLVAMTMGSGAKMKTSASVS |
| 330 | Beta,beta-carotene 9',10'-oxygenase | BCO2 | Q9BYV7 | TSKIRGKAFSDGISWEPQCNTRFHVVEKRTGQLLPGRYYSKPFVTFHQINAFEDQGCVIIDLCCQDNGRTLEVYQLQNL |
| 331 | Cbp/p300-interacting transactivator 1 | CITED1 | Q99966 | MPTTSRPALDVKGGTSPAKEDANQEMSSVAYSNLAVKDRKAVAILHYPGVASNGTKASGAPTSSSGSPIGSPTTTPPTKP |
| 332 | Glyceraldehyde-3-Phosphate Dehydrogenase | GAPDH | P04406 | TVKAENGKLVINGNPITIFQERDPSKIKWGDAGAEYVVESTGVFTTMEKAGAHLQGGAKRVIIS |
| 333 | Retinal-specific ATP-binding cassette transporter | ABCA4 | P78363 | LHPWIYGQQYTFFSMDEPGSEQFTVLADVLLNKPGFGNRCLKEGWLPEYPCGNSTPWKTPSVSPNITQLFQKQKW |
| 334 | Heat shock 70 kDa protein 1AL, 1A, 1B, A2, A8, A6 | HSPA1L; HSPA1A; HSPA1B; HSPA2; HSPA8; HSPA6 | HSPA1A | ACERAKRTLSSSTQASLEIDSL |
| 335 | Cbp/p300-interacting transactivator 1 | CITED1 | Q99966 | LLASMHLQKLNSQYQGMAAATPGQPGEAGPLQNWDFGAQAGGAESLSPSAGAQSPAIIDSDPVDEEV |
| 336 | 60 kDa heat shock protein, mitochondrial | HSPD1 | P10809 | KGVITVKDGKTLNDELEIIEGMKFDRGYISPYFINTSKGQKCEFQDAYVLLSEKKISSIQSIVPALEIANAHRKPLVIIAEDVDGEALSTLVLNRLKVGLQVVAVKAPGFGDNRKNQLKDMAIATGGAVFGEEGL |
| 337 | Myelin basic protein | MBP | P02686 | DENPVVHFFKNIVTPRTPPPSQGKGRGLSLSRFSWGAEGQRPGFGYGGRASDYKSAHKGFKGVDAQGTLSKIFKLGGRDSRSGSPM |
| 338 | Cyclic nucleotide-gated cation channel alpha-3 | CNGA3 | Q16281 | FLLRRWAARHVHHQDQGPDSFPDRFRGAELKEVSSQESNAQANVGSQEPADRGRSAWPLAKCNTNTSNNTEEEK |
| 339 | EGF-containing fibulin-like extracellular matrix protein 1 | EFEMP1 | Q12805 | RRNPADPQRIPSNPSHRIQCAAGYEQSEHNVCQDIDECTAGTHNCRADQVCINLRGSFACQCPPGYQKRG |
| 340 | Protein crumbs homolog 1 | CRB1 | P82279 | NHITLENISSGSSLNVKAGCVRKDWCESQPCQSRGRCINLWLSYQCDCHRPYEGPNCLREYVAGRFGQDDSTGYVIFTLDESYGDTISLSMFVRTL |
| 341 | Tyrosinase | TYR | P14679 | LLCRHKRKQLPEEKQPLLMEKEDYHSLYQSHL |
| 342 | Melanoma antigen recognized by T-cells 1 | MLANA | Q16655 | PREDAHFIYGYPKKGHGHSYTTAEE |
| 343 | X-linked retinitis pigmentosa GTPase regulator-interacting protein 1 | RPGRIP1 | Q96KN7 | YVEYKFYDLPLSETETPVSLRKPRAGEEIHFHFSKVIDLDPQEQQGRRRFLFDMLNGQDPDQGHLKFTVVSDPLDEEKKECEEVGYAYLQLWQILESGRDILEQELDIVSPEDLATPIGRLKVSLQAAAVLHAIYKEMTEDL |
| 344 | Rod cGMP-specific 3',5'-cyclic phosphodiesterase subunit beta | PDE6B | P35913 | MSLSEEQARSFLDQNPDFARQYFGKKLSPENVAAACEDGCPPDCDSLRDLCQVEEST |
| 345 | Retinal-specific ATP-binding cassette transporter | ABCA4 | P78363 | IQSQRKGSEGTCSCSSKGFSTTCPAHVDDLTPEQVLDGDVNELMDVVLHHVPEAKLVECIGQELIFLLPNKNFKHRAYASLFRELEET |
| 346 | Spectrin beta chain, non-erythrocytic 5 | SPTBN5 | Q9NRC6 | TQDHLSQDYESLRALAQLRRARLEEAMALFGFCSSCGELQLWLEKQTVLLQRVQPQADTLEVMQLKYENFLTALAV |
| 347 | Transmembrane protein 98 | TMEM98 | Q9Y2Y6 | IIVVAKRISPRVDDVVKSMYPPLDPKLLDARTTALLLSVSHLVLVTRNACHLTGGLDWIDQSLSAAEEHLEVLREAALASEPDKGLPGPEGFLQEQSAI |
| 348 | Rod cGMP-specific 3',5'-cyclic phosphodiesterase subunit beta | PDE6B | P35913 | DRDEIQLILPTRARLGKEPADCDEDELGEILKEELPGPTTFDIYEFHFSDL |
| 349 | Spectrin beta chain, non-erythrocytic 5 | SPTBN5 | Q9NRC6 | EKVASIALLDLTGARCERLRGRHGRKHTFSLRLTSGAEILFAAPSEEQAESWWRALGSTAAQSLSPKLKAKPVSSL |
| 350 | Protein crumbs homolog 1 | CRB1 | P82279 | LLLALENSTYQYIRVWLERGRLAMLTPNSPKLVVKFVLNDGNVHLISLKIKPYKIELYQSSQNLGFISASTWKIEKGDV |
| 351 | Guanine nucleotide-binding protein G(t) subunit alpha-1 | GNAS; GNAZ; GNA11; GNA15; GNAL; GNA14; GNAQ; GNA13; GNA12; GNAI1; GNAT3; GNAI3; GNAT2; GNAO1; GNAT1; GNAI2 | P11488 | EVKLLLLGAGESGKSTIVKQMKIIH |
| 352 | Retinol dehydrogenase 8 | RDH8 | Q9NYR8 | VQAIVNVISSTRPPLRRQTNIRYSPLTTLKTVDSSGSLYVRTTHRLLFRCPRLLNLGLQCL |
| 353 | Age-related maculopathy susceptibility protein 2 | ARMS2 | P0C7Q2 | KQRSKLSLSHSMIPAAKIHTELCLPAFFSPAGTQRRFQQPQHHLTLSIIHTAAR |
| 354 | Cyclic nucleotide-gated cation channel beta-3 | CNGB3 | Q9NQW8 | NPDPQNAAEPTGTVPEQKEMDPGKEGPNSPQNKPPAAPVINEYADAQLHNLVKRMRQRTALYKKKLVEGDLSSPEASPQTAKPTAVPPVKESDDKPTEHYYRLLWFKVKKMPLTEYLKRIKLPNS |
| 355 | Cyclic nucleotide-gated cation channel alpha-3 | CNGA3 | Q16281 | FARLLAEYNATQMKMKQRLSQLESQVKGGGDKPLADGEVPGDATKTEDK |
| 356 | Retinoschisin | RS1 | O15537 | RLNWIYYKDQTGNNRVFYGNSDRTSTVQNLLRPPIISRF |
| 357 | Rhodopsin kinase | GRK1 | Q15835 | FRARGEKVENKELKHRIISEPVKYPDKFSQASKDFCEALLEKDPEKRLGFRDETCDKLRAHPLFKDLNWRQL |
| 358 | Cyclic nucleotide-gated cation channel beta-1 | CNGB1 | Q14028 | PATKQHPEVQVEDTDADSCPLMAEENPPSTVLPPPSPAKSDTLIVPSSASGTHRKKLPSEDDEAEELKALSPAESPVVA |
| 359 | Glyceraldehyde-3-Phosphate Dehydrogenase | GAPDH | P04406 | EKPAKYDDIKKVVKQASEGPLKGILGYTEHQVVSSDFNSDTHSSTFDAG |
| 360 | cGMP-gated cation channel alpha-1 | CNGA1 | P29973 | ASTSEESENENPHARGSFSYKSLRKGGPSQREQYLPGAIALFNVNNSSNKDQEPEE |
| 361 | RPE-retinal G protein-coupled receptor | RGR | P47804 | SLMEQKLGKSGHLQVNTTLPAR |
| 362 | Interphotoreceptor matrix proteoglycan 1 | IMPG1 | Q17R60 | DTGEYQDWVSICQQETFCLFDIGKNFSNSQEHLDLLQQRIKQRSFPDRKDEISAEKTLGEPGETIVISTDVANVSLGPFPLTPD |
| 363 | Retinoschisin | RS1 | O15537 | SSWTANKARLNSQGFGCAWLSKFQDSSQWLQIDLKEIKVISGILTQGRCDIDEWMTKYSVQYRTDE |
| 364 | Prostaglandin F2-alpha receptor | PTGFR | P43088 | KAVLKNLYKLASQCCGVHVISLHIWELSSIKNSLKVAAISESPVAEKSAS |
| 365 | Cyclic nucleotide-gated cation channel beta-3 | CNGB3 | Q9NQW8 | KGREPEEKPLDRPECTASPIAVEEEPHSVRRTVLPRGTSRQSLIISMAPSAEGGEEVLTIEVKE |
| 366 | cGMP-gated cation channel alpha-1 | CNGA1 | P29973 | GITDMKLSMKNNIINTQQSFVTMPNVIVPDIEKEIRRMENGACSSFSEDDD |
| 367 | Beta,beta-carotene 9',10'-oxygenase | BCO2 | Q9BYV7 | AKSFPRRFVLPLNVSLNAPEGDNLSPLSYTSASAVKQADGTIWCSHENLHQEDLEKEGGIEFPQIYYDRFSGKKYHFFYGCG |
| 368 | EGF-containing fibulin-like extracellular matrix protein 1 | EFEMP1 | Q12805 | NECETTNECREDEMCWNYHGGFRCYPRNPCQDPYILTPENRCVCPVSNAMCRELPQSIVYKYMSIRS |
| 369 | Phakinin | BFSP2 | Q13515 | YVGTAPSGCIGGLGARVTRRALGISSVFLQGLRSSGLATVPAPGLERDHGAVEDLGGCLVEYMAKVHALEQVSQELETQL |
| 370 | Peripherin-2 | PRPH2 | P23942 | LFLKIELRKRSDVMNNSESH |
| 371 | ADP-ribosylation factor-like protein 3 | ARL3 | P36405 | FANKQDLLTAAPASEIAEGLNLHTIRDRVWQIQSCSALT |
| 372 | MAGUK p55 subfamily member 5 | MPP5 | Q8N3R9 | INSGKICLLSLRTQSLKTLRNSDLKPYIIFIAPPSQERLRALLAKEGKNPKPEELREIIEKTREMEQNNGHYFDTAI |
| 373 | Fructose-bisphosphate aldolase A | ALDOA | P04075 | IVAPGKGILAADESTGSIAKRLQSIGTENTEENRRFYRQLLLTADDRVNPCIGGVILFHETLYQKADDGRPFPQVIKSKGGVVGIKVDKGVVPLAGTNGETTTQGL |
| 374 | Fructose-bisphosphate aldolase C | ALDOC | P09972 | MPHSYPALSAEQKKELSDIALRIVAPGKGILAADESVGSMAKRLSQIGVENTEENRRLYRQVLFSADDRVKKCIGGVI |
| 375 | Rod outer segment membrane protein 1 | ROM1 | Q03395 | RYLQTALEGLGGVIDAGGETQGYLFPSGLKDMLKTAWLQGGVACRPAPEEAPPGEAPPKEDLSEA |
| 376 | POU domain, class 4, transcription factor 1 / 2 | POU4F1; POU4F2; POU4F3 | Q01851 / Q12837 | KRKRTSIAAPEKRSLEAYFAIQPRPSSEKIAAIAEKLDLKKNVVR |
| 377 | Rod cGMP-specific 3',5'-cyclic phosphodiesterase subunit alpha | PDE6A | P16499 | EEVEKFLDSNIGFAKQYYNLHYRAKLISDLLGAKEAAVDFSNYHSPSSMEESEIIFDLLRDFQENLQTEKCIFNVM |
| 378 | G protein-coupled receptor kinase 7 | GRK7 | Q8WTQ7 | DPSVVYAKDIAEIDDFSEVRGVEFDDKDKQFFKNFATGAVPIAWQEEIIETGLFEELNDPNRPTGCEEGNSSKSG |
| 379 | Pigment epithelium-derived factor | SERPINF1 | P36955 | KVTQNLTLIEESLTSEFIHDIDRELKTVQAVLTVPKLKLSYEGEVTKSLQEMKLQSLFDSPDFSKITGKPIKLTQVEHRAGFEWNEDG |
| 380 | Protein unc-119 homolog A | UNC119 | Q13432 | MKVKKGGGGAGTATESAPGPSGQSVAPIPQPPAE |
